# No effect of apolipoprotein E polymorphism on MRI brain activity during movie watching

**DOI:** 10.1177/23982128251314577

**Published:** 2025-01-31

**Authors:** Petar P. Raykov, Jessica Daly, Simon E. Fisher, Else Eising, Linda Geerligs, Chris M. Bird

**Affiliations:** 1 Medical Research Council Cognition and Brain Sciences Unit, University of Cambridge, Cambridge, UK; 2 School of Psychology, University of Sussex, Falmer, UK; 3 Language and Genetics Department, Max Planck Institute for Psycholinguistics, Nijmegen, The Netherlands; 4 Donders Institute for Brain, Cognition and Behaviour, Radboud University, Nijmegen, The Netherlands

**Keywords:** Cognition, apolipoprotein E, naturalistic fMRI, aging, functional segregation, event boundary

## Abstract

Apolipoprotein E ε4 is a major genetic risk factor for Alzheimer’s disease, and some apolipoprotein E ε4 carriers show Alzheimer’s disease–related neuropathology many years before cognitive changes are apparent. Therefore, studying healthy apolipoprotein E genotyped individuals offers an opportunity to investigate the earliest changes in brain measures that may signal the presence of disease-related processes. For example, subtle changes in functional magnetic resonance imaging functional connectivity, particularly within the default mode network, have been described when comparing healthy ε4 carriers to ε3 carriers. Similarly, very mild impairments of episodic memory have also been documented in healthy apolipoprotein E ε4 carriers. Here, we use a naturalistic activity (movie watching), and a marker of episodic memory encoding (transient changes in functional magnetic resonance imaging activity and functional connectivity around so-called ‘event boundaries’), to investigate potential phenotype differences associated with the apolipoprotein E ε4 genotype in a large sample of healthy adults. Using Bayes factor analyses, we found strong evidence against existence of differences associated with apolipoprotein E allelic status. Similarly, we did not find apolipoprotein E-associated differences when we ran exploratory analyses examining: functional system segregation across the whole brain, and connectivity within the default mode network. We conclude that apolipoprotein E genotype has little or no effect on how ongoing experiences are processed in healthy adults. The mild phenotype differences observed in some studies may reflect early effects of Alzheimer’s disease–related pathology in apolipoprotein E ε4 carriers.

## Introduction

Carrying the ε4 allele of the apolipoprotein E (APOE) gene is a significant risk factor for late-onset sporadic Alzheimer’s disease (AD) (Genin et al., 2011). In healthy adults, the ε4 allele has also been associated with poorer cognitive abilities and steeper decline of episodic memory with age (Henson et al., 2020; Jack et al., 2015; Jochemsen et al., 2012; Lyall et al., 2016, 2019; Marioni et al., 2016; Mondadori et al., 2007; Schiepers et al., 2012; Schultz et al., 2008; Shin et al., 2014; Wisdom et al., 2011). Furthermore, several studies have reported atypical functional magnetic resonance imaging (fMRI) brain activity in healthy adult ε4 carriers compared with non-carriers. For example, increased activation in medial temporal lobe has been observed during memory tasks (Brunec et al., 2020b; Trelle et al., 2020), and increased functional connectivity has been described throughout the default mode network (DMN) during resting state scans (Filippini et al., 2009; Henson et al., 2020; Hodgetts et al., 2019; Su et al., 2015; Trachtenberg et al., 2012; Westlye et al., 2011). Interestingly, these regions are strongly associated with ‘event processing’, our ability to both understand our experiences in the moment and remember them later (Bird, 2020; Bromis et al., 2022; Chen et al., 2017; Raykov et al., 2021; Stawarczyk et al., 2021). Taken together, these findings suggest a potential link between APOE-related alterations in brain function and behavioural changes in encoding and retrieving memories of personally experienced events.

In the present study, we investigated a marker of long-term memory encoding: the transient fMRI responses to ‘event boundaries’ while people watch movies (Baldassano et al., 2017; Radvansky and Zacks, 2017; Reagh et al., 2020; Zacks et al., 2007). In narrative materials, such as stories or movies, event boundaries coincide with the conclusion of a piece of action and the beginning of another. Importantly, fMRI activity in the hippocampus and DMN at event boundaries has been found to predict later memory for the events, decrease with age and to coincide with increased functional connectivity in DMN (Barnett et al., 2024; Ben-Yakov et al., 2013; Ben-Yakov and Henson, 2018; Bird, 2020; Cooper et al., 2021; Reagh et al., 2020).

Overall, APOE ε4 carriers perform very slightly worse than non-carriers when tested on traditional episodic memory tests using word or pictorial memoranda (e.g. d = -0.14; Wisdom et al., 2011). However, there have been no studies investigating the effect of APOE genotype on event processing and memory using narrative-based materials, which may better capture the cognitive processes that are in play during our everyday experiences, compared to clinic-based memory assessments (Bird, 2020; Jolly and Chang, 2019; Matusz et al., 2019). There is extensive evidence that events are processed differently across the lifespan and in the context of mild AD (Bailey et al., 2013, 2015; Jekel et al., 2015; Petersen, 2000; Roll et al., 2019; Zacks et al., 2006). For example, individuals with mild AD tended to be more variable in where they place event boundaries and overall put fewer event boundaries than healthy individuals (Zacks et al., 2006). Moreover, this inefficiency in ‘segmenting’ experience at event boundaries is both related to reduced hippocampal volume and ability to remember the events (Bailey et al., 2013). Given the association between APOE ε4 genotype and AD-related brain changes, we predicted that brain activity indices of event processing and event memory encoding might be modulated by APOE status in a large sample of healthy adults. To test this, we analysed data from the Cambridge Centre for Ageing and Neuroscience (Cam-CAN) data repository. In our main pre-registered analyses, we focused on examining genotype-related differences in measures that have been strongly associated with event processing – changes in univariate activity and connectivity among the DMN regions at event boundaries. Because we did not observe any genotype differences, we also ran exploratory analyses using multivariate classification to examine whether patterns of DMN connectivity at event boundaries contained information that could distinguish different APOE carriers. In addition, we explored whether functional connectivity computed across the whole duration of the movie and summarised across the whole brain using a measure of functional system segregation showed genotype-associated difference. We did not find strong evidence for APOE effects across any of our measures.

## Methods

### Participants

**Figure 1.**
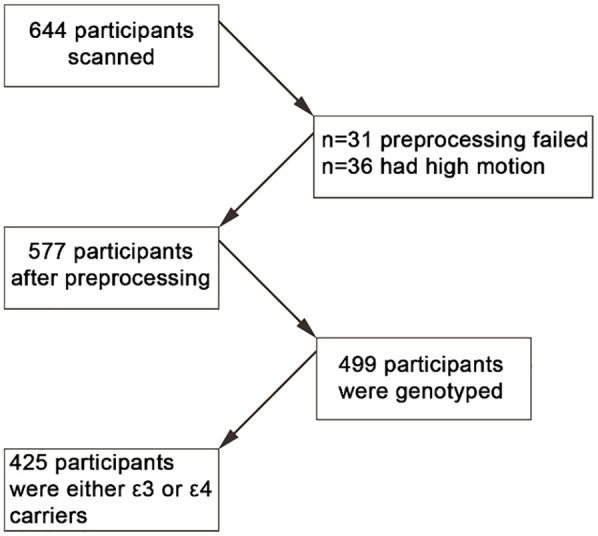
Flow chart of participants’ selection. Figure shows selection of participants to be included in final analysis.

A total of 644 participants (18–88 years old, M = 54.3, standard deviation [SD] = 18.46, 318 males and 326 females) were included in this study, from the population-based sample of Cam-CAN. Of the initial sample, 577 participants were considered for further analyses due to various issues with data quality or pre-processing (see Figure 1). For example, 31 participants were excluded due to failure to run spatial normalisation or ME-ICA denoising. In addition, 36 participants were excluded due to high motion. Here, we used already pre-processed fMRI data from the movie task in the Cam-CAN cohort as described in a previous report (Geerligs and Campbell, 2018). The mean age of the 577 included participants was 53.39 ± 18.42 (18–88). Participants were included if no brain abnormalities were detected and if they completed the full (f)MRI testing session. Participants scored 25 or higher on the mini mental state examination (Folstein et al., 1975), did not have a current diagnosis of dementia or mild-cognitive impairment, had normal or corrected-to-normal vision and hearing, were native English speakers, and had no neurological disorders. Furthermore, we only included participants for whom we had APOE genotype data (n = 499). Given the small prevalence of the ε2 allele variant, we only included participants who were carriers of the ε3 and ε4 alleles. Participants were grouped into a single ε4+ group (ε4/ε3 and ε4/ε4) or an ε3 group (ε3/ε3). This resulted in 425 participants: 134 people in the ε4+ group (126 were ε4/ε3 and 8 were ε4/ε4) and 291 in the ε3 group (all ε3/ε3). The participants had a mean age of 53.83 years with standard deviation of 18.28. Ethical approval for the study was obtained from the Cambridgeshire 2 (now East of England-Cambridge Central) Research Ethics Committee. Participants gave written informed consent.

### Materials

Participants were scanned while watching a shortened version of the Alfred Hitchcock movie called ‘Bang! You’re Dead’. The movie lasted 8 min with the narrative of the episode preserved (Shafto et al., 2014). Participants were instructed to pay attention to the movie (they were not made aware of its title, and no participants reported having seen it before).

### fMRI data and image acquisition

Data were collected as a part of more extensive scanning session on a 3T Siemens TIM Trio, with a 32-channel head coil. Full details about the scanning protocol can be found in Shafto et al. (2014). Data for the movie task were collected using multi-echo T2*-weighted echo-planar imaging (EPI) sequence. Each volume contained 32 axial slices (acquired in descending order), with slice thickness of 3.7 mm and interslice gap of 20% (repetition time (TR) = 2470 ms; 5 echoes [echo time (TE) = 9.4 ms, 21.2 ms, 33 ms, 45 ms, 57 ms]; flip angle = 78°; field-of-view (FOV) = 192 mm × 192 mm; voxel size 3 mm × 3 mm × 4.44 mm). Acquisition time was 8 min and 13 s and this resulted in 193 volumes.

In addition, a high-resolution (1 mm × 1 mm × 1 mm) T1-weighted Magnetization Prepared Rapid Gradient Echo image was acquired, in addition to a T2-weighted structural image (voxel size 1 mm × 1 mm × 1 mm).

### Functional data pre-processing

Data were pre-processed using AFNI (version AFNI_17.1.01; https://afni.nimh.nih.gov) and SPM12 (http://www.fil.ion.ucl.ac.uk/spm) as implemented in the automatic analysis (AA) batching system. This enabled the use of multi-echo independent component analysis (ME-ICA) carried out in AFNI and DARTEL interparticipant alignment in SPM, which allows for transformation to an age-representative template that is subsequently transformed to Montreal Neurological Institute (MNI) space. ME-ICA tries to separate Blood Oxygenation Level Dependent (BOLD)-related components from non-BOLD-like components in the data and has been shown as a useful method to reduce false positives in both rest (Kundu et al., 2012) and task data (Gonzalez-Castillo et al., 2016). The ME-ICA was fit using the meica.py algorithm (Kundu et al., 2012), which performs slice-timing correction, and motion realignment on the individual TE images. This is followed by applying Principal Component Analysis (PCA) and afterwards FastICA in order to decompose the data into spatially independent components, which are characterised as BOLD-like (high-kappa) components and non-BOLD-like (low-kappa) components. The imaging data is reconstructed using only the BOLD-like components. After normalisation, the data were smoothed with an 8-mm full width half at maximum (FWHM) kernel.

Region of Interest (ROI) definition was based on the Craddock atlas and a hippocampal segmentation based on Ritchey et al. (2015). Signals were extracted from 748 of the 840 regions defined by Craddock et al. (2012), because previously they were identified to have sufficient coverage (Geerligs et al., 2015). In addition, we extracted signal from four hippocampal ROIs. Specifically, signal was extracted from the left and right anterior hippocampus and left and right posterior hippocampus (combined body and tail from https://neurovault.org/collections/3731/).

Apart from ME-ICA de-noising, a General Linear Model (GLM) was applied to the averaged ROI signal to reduce residual motion effects. Specifically, for each ROI, the model included the six original motion parameters, as well as the timepoint-by-timepoint average of the white matter (WM) and cerebrospinal fluid (CSF) signals. The WM and CSF signals were computed from masks that had less than 1% chance to contain grey matter and had more than 80% tissue probability value of WM/CSF. Additional to these eight confound regressors, the model included their temporal derivatives, their squares, and the squared derivatives. In total, there were 32 confound and noise regressors. A high-pass filter (0.008 Hz) was implemented by including a discrete cosine transform set in the GLM, ensuring that nuisance regression and filtering were performed simultaneously. To account for autocorrelation in the signal, we fitted a model of eight exponentials with half-lives from 0.5 to 64 TRs to the GLM error. The whitened residuals of this model were used for all analyses reported in the paper. For full details of the pre-processing procedure, see Geerligs and Campbell (2018).

### Data analysis

All analyses were performed as pre-registered (https://osf.io/un5wa). Analyses were done in MATLAB and R using adapted scripts from Henson et al. (2020; see https://osf.io/ehs9n/). For all analyses, we focused on the 425 people with available APOE ε4/ε3 genotype data (134 ε4+ and 291 ε3 carriers).

### Event boundary functional activation

Our main interest was examining whether BOLD activity during event boundaries in the movie showed differences associated with APOE allelic status. In a previous study (Ben-Yakov and Henson, 2018), an independent sample of 16 participants were asked to watch the movie and indicate when they perceived an event boundary had occurred. Participants had to indicate when they felt one meaningful event ended and another started. This resulted in 19 event boundaries identified across participants. Previous work has focused on event boundaries consistently identified by at least 50% of the 16 participants, leaving us with 12 event boundaries (see Ben-Yakov and Henson, 2018; Cooper et al., 2021; Reagh et al., 2020). Reagh et al. (2020) ran an additional behavioural segmentation experiment using the Cam-CAN movie showing that older adults exhibited similar event segmentation behaviour to younger adults.

Our main dependent variables were the univariate activity during event boundaries in hippocampal and posterior DMN regions. We focused on these regions since previous research has found both univariate responses and changes in connectivity in response to event boundaries (Cooper et al., 2021; Reagh et al., 2020). Specifically, we included the left and right anterior and posterior hippocampus and posterior DMN regions such as retrosplenial cortex (RSC), extending to posterior medial cortex (PMC) and left and right angular gyrus (AG).

### Event boundary functional connectivity

To examine event boundary functional connectivity, we used a previous approach that reported that posterior DMN regions showed connectivity differences during event boundaries compared to within-event time-points (Cooper et al., 2021). Specifically, we first converted the event boundary onsets to a binarized vector matching the whole duration of the movie task. The vector was 0 s for all within-event time-points and 1 s whenever an event boundary occurred. The onsets, in seconds, were shifted by 2 TRs (~5 s) to account for the hemodynamic lag. In order to take into consideration the transition in connectivity from one event to the next, we also included 2 TRs around the event boundary onsets. For instance, if an event boundary occurred 30 s into the movie, we would model the 25.06 s up to 34.94 s as the event transition period and then shift it by 2 TRs to account for the hemodynamic lag.

For each pair of ROIs, we than constructed a logistic regression model that predicted the event boundary transition period from the average activity in ROI_1_, ROI_2_, and their interaction (coactivation) [event boundaries ~ ROI_1_ + ROI_2_ + ROI_1_ × ROI_2_]. The beta coefficient for the interaction reflects the change in coactivation between the pair of ROIs during event boundaries controlling for the activity time series of each ROI, similar to psycho physiological interaction (PPI) analysis (see Faskowitz et al., 2020). We used these event boundary-related coactivation matrices to define the ROIs used in the main phenotype analyses. Critically, we then examined whether coactivation patterns differed between ε3/ε3 homozygotes and ε4 carriers.

### ROI definition

**Figure 2.**
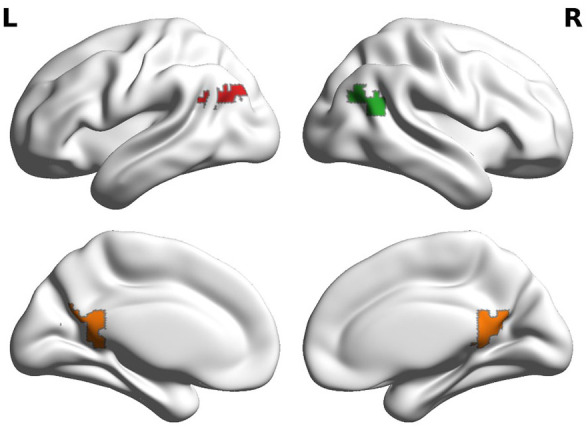
Craddock ROIs used in analyses. We defined regions with similar functional coactivation during event boundaries from the DMN. We additionally examined whether APOE genotype was associated with differences in the hippocampus (not shown).

As pre-registered (https://osf.io/un5wa), we focused on posterior regions of the DMN extracted from the Craddock et al. (2012) functional atlas. Before analysis of any APOE genotype-associated differences, we computed how functional connectivity changed at event boundaries across these posterior DMN regions. Recent work has suggested that different sub-regions of the DMN change their connectivity differently in response to the event boundaries (Cooper et al., 2021). After computing the event boundary functional connectivity, we focused on Craddock regions that were contiguous and all increased their connectivity among each other and to the hippocampus during event boundaries when compared to within-event time-points. The increased functional connectivity suggested that these regions might act as a single functional unit. This resulted in a left AG, right AG, and PMC region that were constructed from smaller Craddock regions (see Figure 2). The ROI definition was implemented and pre-registered by the author P.P.R. before the genotype data was made available. The combined left AG region consisted of Craddock regions labelled 730, 675, and 664. The combined right AG region was constructed from Craddock regions 680, 728, and 669; and a combined RSC/PMC region consisting of Craddock regions 725, 699, 703, 710, and 726. We did not observe functional connectivity differences among the hippocampal sub-regions, so we combined the four hippocampal regions into a single hippocampal ROI.

### Statistical models

For each of the univariate and coactivation-dependent variables, we examined the effects of APOE and age. We fitted linear models that included a linear and a quadratic effect of age (y ~ ß_0_ + ß_1_ × age + ß_2_ × age^2^). All regressors of interest were Z-scored. We removed outliers on any of the dependent variables that were defined by residuals that were 1.5 times the interquartile range, after adjusting for polynomial effects of age. The pipeline for the models used here was pre-registered and was adapted from a previous pre-registered report (Henson et al., 2020).

For each dependent variable, we modelled the effects of age separately for the two genotype groups (ε3 carriers versus ε4 carriers). We had planned comparisons comparing whether age effects differed in ε3 versus ε4 carriers. We ran two-tailed t-tests examining the main effect of APOE, APOE by linear effect of age, and APOE by quadratic age effect interactions. We report uncorrected p-values since nothing was significant even before applying multiple-comparisons correction.

Apart from frequentist statistics, we also report Bayes factors calculated for each effect. We fitted the same linear models as above with the brms package (Bürkner, 2017) using 100,000 Markov chain Monte Carlo (MCMC) iterations.

## Results

### Main effects for functional activation and connectivity

#### Univariate activation (see Fig. 3)

**Figure 3.**
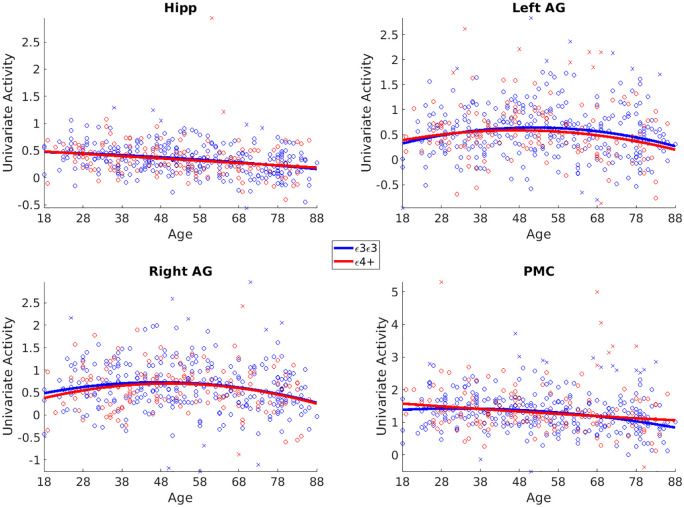
Univariate results. There were no genotype effects on univariate BOLD activity at event boundaries during movie watching. Scatter plots for each ROI against age grouped by APOE genotypes (ε3/ε3 homozygotes and ε4 carriers). Outliers are indicated by crosses and were not included in the final analyses. The solid lines indicate quadratic fits of age for each genotype group.

Before examining phenotype effects, we examined main effects of event boundaries on univariate activity and functional connectivity. Using a different pre-processing strategy from Reagh and Ranganath (2023), we replicated their previous results showing higher BOLD signal during event boundaries in bilateral hippocampus (t_424_ = 23.98, p < 0.001), left and right AG (t_424_ = 21.21, p < 0.001; t_424_ = 23.46, p < 0.001), and RSC/PMC (t_424_ = 41.32, p < 0.001) regions (see Figure 3). We also replicated Reagh and Ranganath’s results of decreased activation in old age in bilateral hippocampus (Linear Age β = −0.08, t = −6.20, p < 0.001). We additionally found linear age effects on boundary activation in PMC (Lin Age β = −0.13, t = −5.26, p < 0.001) and linear and/or quadratic age effects on left (Lin Age β = −0.03, t = −1.57, p = 0.11; Quad Age β = −0.08, t = −3.22, p = 0.001) and right (Lin Age β = −0.05, t = −2.1, p = 0.03; Quad Age β = −0.09, t = −3.78, p < 0.001) AG.

#### Functional connectivity (see Fig. 4)

**Figure 4.**
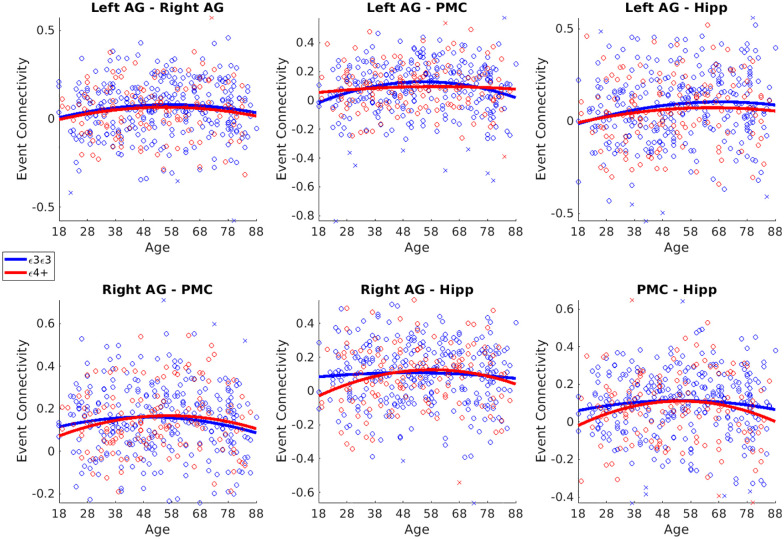
Coactivation results. There were no genotype effects on event locked functional coactivation between pairs of ROIs in the DMN. Scatter plots for each ROI against age grouped by APOE genotypes (ε3/ε3 homozygotes and ε4 carriers). Outliers are indicated by crosses and were not included in the final analyses. The solid lines indicate quadratic fits of age for each genotype group.

We observed increased functional coactivation among DMN regions and used this as a criterion to define our ROIs. This increased DMN coactivation replicates and extends previous results in Cooper et al. (2021), where they focused on a sub-sample of Cam-CAN participants. Across the whole age range, we did not observe a strong decrease in pairwise connectivity in the selected regions (see Figure 4). Left to Right AG (Lin Age β < 0.01, t = 0.6, p = 0.52; Quad Age β = −0.01, t = −1.90, p = 0.05); Left AG to PMC (Lin Age β = 0.05, t = 0.7, p = 0.48; **Quad Age β** **=** −**0.02, t** **=** −**2.48, p** **=** **0.01**); Left AG to Hipp (**Lin Age β** **=** **0.02, t** **=** **2.2, p** **=** **0.02**; Quad Age β = −0.01, t = −1.36, p = 0.17); Right AG to PMC (Lin Age β < 0.01, t = −0.06, p = 0.95; **Quad Age β** **=** −**0.02, t** **=** −**2.14, p** **=** **0.03**); Right AG to Hipp (Lin Age β = 0.01, t = 0.75, p = 0.45; **Quad Age β** **=** −**0.02, t** **=** −**1.97, p** **=** **0.049**); PMC to Hipp (Lin Age β < 0.01, t = 0.29, p = 0.77; **Quad Age β** **=** −**0.02, t** **=** −**2.46, p** **=** **0.014**).

### APOE effects for Event Boundaries functional activation and connectivity

We examined whether there were APOE-related differences in univariate and connectivity measures during event boundaries in hippocampus and posterior DMN regions. We found little evidence that differences in any of our dependent variables were associated with APOE allelic status (ε3/ε3 homozygotes versus ε4 carriers). For the univariate BOLD activity at event boundaries, we found no differences associated with genotype, or genotype by age interactions. Indeed, Bayes factors provided strong evidence in support of null effects (see Figure 3 and Table 1).

**Table 1.** GLM results for APOE effects for univariate activity at movie boundary in each ROI.

ε4+ versus ε3/ε3				
	Fit	0 Main APOE effect	1 Linear age by APOE	2 Quadratic age by APOE
Hipp	**R**^2^ **=** **0.66** **df** **=** **411**	-0.0001 (0.012) T = -0.14 p = 0.98 BF_01_ = 21.23	0.003 (0.012) T = 0.27 p = 0.78 BF_01_ = 19.22	0.005 (0.012) T = 0.39 p = 0.69 BF_01_ = 18.18
Left AG	**R**^2^ **=** **0.58** **df** **=** **401**	-0.018 (0.022) T = -0.79 p = 0.42 BF_01_ = 14.86	-0.012 (0.024) T = -0.52 p = 0.60 BF_01_ = 16.45	0.007 (0.24) T = 0.29 p = 0.77 BF_01_ = 18.16
Right AG	**R**^2^ **=** **0.64** **df** **=** **407**	-0.01 (0.022) T = -0.47 p = 0.63 BF_01_ = 18.44	0.01 (0.024) T = 0.65 p = 0.51 BF_01_ = 15.95	-0.004 (0.024) T = -0.16 p = 0.86 BF_01_ = 19.12
PMC	**R**^2^ **=** **0.87** **df** **=** **396**	0.019 (0.024) T = 0.80 p = 0.42 BF_01_ = 14.93	0.009 (0.026) T = 0.37 p = 0.71 BF_01_ = 17.83	0.03 (0.026) T = 1.23 p = 0.21 BF_01_ = 9.05

Hipp: hippocampus; AG: angular gyrus; PMC: posterior medial cortex; R^2^: adjusted R-squared for full mode; df: degrees of freedom; BF_01_: Bayes factor in support of null hypothesis of no effect. First column shows full model fit. The following columns show the parameter estimate, standard error, T-statistic, p-value and Bayes factor, for the APOE by age interaction effects. Significant effects are shown in bold.

In addition, when we examined whether functional coactivation during event boundaries differed, we did not observe any genotypic associations or genotype by age interactions in either linear or quadratic terms (see Figure 4 and Table 2). Bayes factors showed evidence for the null hypotheses of no differences between the ε3/ε3 homozygotes and ε4 carriers.

**Table 2.** GLM results for APOE effects for functional coactivation at movie boundary in each ROI.

ε4+ versus ε3/ε3				
	Fit	0 Main APOE effect	1 Linear age by APOE	2 Quadratic age by APOE
L AG – Hipp	**R**^2^ **=** **0.15** **df** **=** **413**	-0.01 (0.008) T = -1.32 p = 0.18 BF_01_ = 8.73	-0.004 (0.009) T = -0.49 p = 0.62 BF_01_ = 17.17	0.001 (0.009) T = 0.18 p = 0.85 BF_01_ = 18.88
L AG – R AG	**R**^2^ **=** **0.13** **df** **=** **415**	-0.006 (0.007) T = -0.91 p = 0.35 BF_01_ = 13.62	-0.000 (0.007) T = -0.039 p = 0.96 BF_01_ = 19.04	-0.000 (0.007) T = -0.001 p = 0.99 BF_01_ = 18.89
L AG – PMC	**R**^2^ **=** **0.28** **df** **=** **408**	-0.003 (0.007) T = -0.48 p = 0.62 BF_01_ = 18.17	0.0003 (0.007) T = 0.048 p = 0.96 BF_01_ = 19.13	0.012 (0.007) T = 1.6 p = 0.11 BF_01_ = 5.32
R AG – PMC	**R**^2^ **=** **0.46** **df** **=** **416**	0.01 (0.007) T = 0.14 p = 0.88 BF_01_ = 20.55	0.008 (0.008) T = 1.0 p = 0.27 BF_01_ = 10.63	-0.001 (0.008) T = -0.24 p = 0.80 BF_01_ = 18.44
R AG – Hipp	**R**^2^ **=** **0.23** **df** **=** **416**	-0.003 (0.008) T = -0.45 p = 0.65 BF_01_ = 18.63	0.01 (0.009) T = 1.09 p = 0.27 BF_01_ = 10.49	-0.01 (0.009) T = -1.18 p = 0.23 BF_01_ = 9.59
PMC – Hipp	**R**^2^ **=** **0.24** **df** **=** **410**	-0.009 (0.008) T = -1.17 p = 0.24 BF_01_ = 10.20	0.002 (0.008) T = 0.28 p = 0.77 BF_01_ = 18.03	-0.008 (0.008) T = -0.95 p = 0.33 BF_01_ = 12.13

Hipp: hippocampus; AG: angular gyrus; PMC: posterior medial cortex; R^2^: adjusted R-squared for full mode; df: degrees of freedom; BF_01_: Bayes factor in support of null hypothesis of no effect. First column shows full model fit. The following columns show the parameter estimate, standard error, T-statistic, p-value and Bayes factor, for the APOE by age interaction effects. Significant effects are shown in bold.

### Exploratory classification analyses

**Figure 5.**
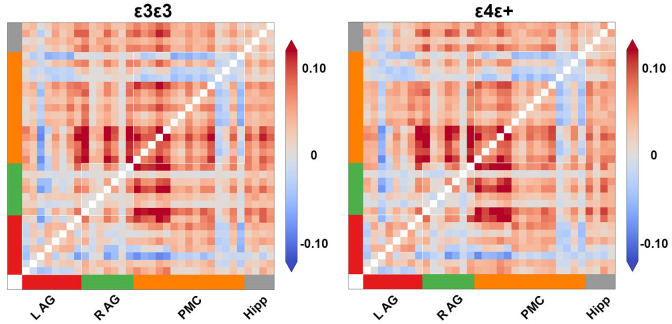
Pairwise coactivation matrices per genotype group. The figure shows the average pairwise coactivation matrix during EBs for all posterior Craddock DMN regions and the four hippocampal sub-region ROIs per genotype group. We note we could not classify participant’s genotype when using these matrices as features in multivariate across person classification analyses. AG: angular gyrus; PMC: posterior medial cortex; Hipp: hippocampus; L: left; R: right.

To examine whether there was some information present in the pattern of functional connectivity that could differentiate between the APOE allelic groups within our sample, we ran exploratory multivariate classification analyses in Supplementary Materials. We ran support vector machine (Cortes and Vapnik, 1995; for overview see Raykov and Saad, 2022) as implemented in scikit-learn (Pedregosa et al., 2011), where we used the full coactivation matrices, as defined by the PPI-like analysis, as features and tried to predict participant’s genotype. We show averaged pairwise coactivation matrices per genotype group for all posterior DMN regions and the four sub-regions of the hippocampus in Figure 5. We used balanced accuracy measure and stratified cross validation to account for class imbalance (see Thölke et al., 2023). This analysis ignored genotype by age interactions and aimed to utilise multivariate information that may be present in the full coactivation matrix that we might have missed by focusing on a smaller number of posterior DMN regions. To test significance, we ran classification analyses 5000 times with randomly permuted genotype labels. We did not find significant classifier performance above chance (bAcc = 0.54, p = 0.64).

### Exploratory DMN and whole-brain functional connectivity analyses

In Supplementary results, we also report exploratory analyses where we examined (1) average DMN connectivity measures across the whole duration of the movie and (2) a graph theoretical measure of global connectivity across the whole brain computed for the full duration of the movie. We first tested whether APOE allelic status was associated with differences in average connectivity among all DMN regions during movie watching as has been previously found during resting state in the same cohort (Henson et al., 2020). We did not observe a main effect of genotype, and the Bayes factor was in support of the null hypothesis. The Bayes factor for the linear age by genotype interaction was also in support of the null, but we note that it was the effect with lowest evidence for the null in this report.

Since our main analyses focused only on sub-regions of the DMN and may have missed effects that are more widespread, we wanted to examine whole-brain connectivity measures. Multiple previous reports have shown that age is associated with decrease in within-network connectivity and increase in between network connectivity, a pattern of connectivity often termed as ‘functional system segregation’ (SyS) (Chan et al., 2014; Deery et al., 2023; Pedersen et al., 2021; Raykov et al., 2024; Tsvetanov et al., 2016). Higher SyS has been associated with better memory and fluid intelligence, and is crucial for optimal brain function (Chan et al., 2014; Raykov et al., 2024; Wig, 2017). Interestingly, recently it has been shown that higher SyS can attenuate the relationship between AD severity and cognition (Ewers et al., 2021). Furthermore, a recent study has shown that ε4 carriers showed accelerated decrease in SyS over the course of 2 years compared to non-carriers (Deng et al., 2023).

In a prior report (Raykov et al., 2024), we had computed SyS values for the Cam-CAN cohort using the Craddock functional atlas (Craddock et al., 2012) and previously defined age-representative networks (Geerligs et al., 2015). As in previous work, we computed SyS across regions in part of associative brain networks, after performing global signal regression to account for vascular effects. The SyS measure was computed as the difference of within and between network connectivity scaled by within-network connectivity. We did not observe any differences in SyS measures between ε3/ε3 homozygotes and ε4 carriers. Bayes factors again favoured the null of no differences associated with allelic status.

## Discussion

Here, we used the Movie task from the Cam-CAN dataset to test whether there are APOE-related differences in biological markers of memory encoding: the transient fMRI responses to event boundaries within the hippocampus and DMN regions. Specifically, we compared APOE ε4 carriers to ε3/ε3 homozygotes and investigated both the univariate response within these brain regions as well as functional connectivity changes around event boundaries. We found no evidence for differential activity or connectivity at event boundaries in either the hippocampus or the DMN network, across ε4 carriers versus ε3/ε3 homozygotes. Moreover, there were no genotype by age interactions. In addition to our pre-registered analyses, we ran exploratory analyses: examining multivariate connectivity patterns at event boundaries; examining connectivity within the DMN across the whole movie; and computing whole-brain measure of functional system segregation. None of these exploratory analyses showed differences associated with the APOE genotypes tested in our sample.

We did not support our main hypothesis that APOE status would modulate fMRI activity and connectivity measures around event boundaries. This was a surprising result for at least three reasons. First, episodic memory impairments in APOE ε4 carriers are often reported, and the hippocampal fMRI response to event boundaries has been thought to index episodic memory encoding (Ben-Yakov and Henson, 2018; Reagh et al., 2020). Second, within the same participant cohort studied here, the APOE ε4 carriers had lower functional connectivity within the DMN during their resting state fMRI scan (Henson et al., 2020), and event boundaries during movie watching have been associated with functional activation of this network. Third, some studies have associated APOE ε4 carriers with altered processing of spatial boundaries during navigation tasks (Alkan et al., 2021; Bierbrauer et al., 2020; Colmant et al., 2023), and spatial boundaries have been suggested to be similar to event boundaries (Brunec et al., 2020a). We will discuss the implications of our study below.

Meta-analyses of the effects of APOE status on episodic memory function have shown very small (ds −0.01 to −0.14), but sometimes significant, impairments in APOE ε4 carriers (Lancaster et al., 2017; Small et al., 2004; Tank et al., 2021; Wisdom et al., 2011). Consistent with this, several studies of large cohorts of participants have documented APOE-related differences in episodic memory performance (Tank et al., 2021) or faster decline of episodic memory in ε4 carriers, compared with ε3 homozygous individuals (Caselli et al., 2009; Corley et al., 2023; Rawle et al., 2018). However, it is important to note that differences in the rate of decline are often only seen in adults aged 60 or 70 years old or may only be present in ε4 homozygous individuals. Moreover, a recent cohort study which followed up participants for up to 6 years to verify none received a diagnosis of dementia found no effect of APOE on episodic memory (Kauppi et al., 2020). The Cam-CAN cohort was aged between 18 and 89 years, was neurologically healthy, and there were no differences on the logical memory test of verbal recall associated with APOE allelic status (Henson et al., 2020). While some studies have reported APOE-related functional brain changes in the absence of behavioural effects (Evans et al., 2018; Green et al., 2014; Kunz et al., 2015), our results suggest no differences between healthy APOE ε4 carriers and ε3/ε3 homozygotes in episodic memory encoding when carrying out naturalistic tasks such as movie watching.

A previous investigation of resting state connectivity in the same cohort of participants as the present study found increased functional connectivity within the DMN in APOE ε4 carriers (Henson et al., 2020). Moreover, there is considerable evidence that the DMN is engaged during movie watching in general, and at event boundaries in particular (Baldassano et al., 2017; Ben-Yakov and Henson, 2018; Bird, 2020; Chen et al., 2017; Cooper et al., 2021; Raykov et al., 2021; Reagh and Ranganath, 2023). We therefore predicted that the coactivation of DMN regions around event boundaries would be modulated by APOE status. However, this hypothesis was not supported. We note that while some studies have also reported increased DMN functional connectivity during resting state scans in APOE ε4 carriers (Filippini et al., 2009; Westlye et al., 2011), others have reported the opposite effect (Kucikova et al., 2023; Machulda et al., 2011; Mentink et al., 2021) or the results have been mixed (Damoiseaux et al., 2012; Sheline et al., 2010), or found no differences (Dowell et al., 2016; Mentink et al., 2021). In our main analyses, we focused on functional connectivity at event boundaries, whereas resting state functional connectivity is often computed through the whole session. In post hoc analyses, we computed connectivity across the whole movie duration and summarised the connectivity using graph theoretical measure of system segregation (SyS). SyS has been shown to decline with age and to be related to cognition (Chan et al., 2014; Deery et al., 2023; Pedersen et al., 2021; Raykov et al., 2024; Wig, 2017) and APOE status (Deng et al., 2023). We did not observe any difference in SyS measures across APOE carriers. Our study shows that functional connectivity across the DMN during movie watching is not modulated by APOE status, even in a cohort that shows DMN functional connectivity differences during rest. It may be that the activity of movie watching drives neural activity in similar ways across genotypes, whereas the unconstrained nature of resting state scanning is more sensitive to genotype-related differences.

The last reason for predicting that the processing of event boundaries might be modulated by APOE status is the finding that spatial boundaries are processed differently in APOE ε4 carriers, compared to non-carriers (Alkan et al., 2021; Colmant et al., 2023; Kunz et al., 2015). One clear-cut difference between our study and that of Kunz and colleagues is that our study involves the incidental encoding of information while engaged in an everyday activity – movie watching – rather than a memory test. Consequently, it may be that processing of both spatial and event boundaries are affected by APOE status, but that this is only apparent during effortful tasks. Processing event boundaries in movies may also differ from processing spatial boundaries because understanding the movie’s narrative is strongly supported by prior knowledge (Baldassano et al., 2017; Bartlett, 1932; Masís-Obando et al., 2022; Raykov et al., 2021, 2022, 2023; Silva et al., 2019). Umanath and Marsh (2014) have argued that the ability to scaffold new learning with pre-existing knowledge may enable older adults to compensate for episodic memory decline. A final putative explanation for the difference between our study and those that analysed spatial boundaries is that the processing of spatial boundaries is functionally separable from event processing and might be preferentially modulated by APOE status. Certainly, some have argued that spatial processing might be particularly sensitive to early AD-related pathology (Coughlan et al., 2018, 2019; Gellersen et al., 2021), although overall, the effects of APOE status on spatial processing are extremely small (Daly et al., 2024).

It is still unclear through what mechanisms the ε4 allele increases risk of developing AD. Recent work has suggested that carrying the ε4 allele can result in increased presence of white-matter hyperintensities (Heise et al., 2024; Lyall et al., 2020), which may be an indicator of poorer cardiovascular health. Work using animal models has suggested that the ε4 allele may have multiple effects on cell and brain health that each contribute to increasing brain vulnerability to pathology (Steele et al., 2022). However, it is still not clear whether ε4 affects cognition before development of AD pathology (e.g. Vemuri et al., 2010). Indeed, a recent large-scale cross-sectional study found that APOE had very little impact on cognitive functioning in healthy aging suggesting that genetic and cellular mechanisms linked to dementia risk may be distinct from the mechanisms involved in healthy cognitive aging (Rahman et al., 2024).

Finally, we note that our study had several limitations. The study could have suffered from selection bias specifically for older individuals, as participants were excluded if they showed evidence of cognitive decline. Indeed, this could have affected our estimates as we may have excluded individuals with early stages of AD pathology (Vemuri et al., 2010). Further limitation was that we focused on a single gene that increases the risk of dementia rather than examining polygenic risk factors. Although studying single genes in the general population can offer valuable insights into the biological mechanisms underlying brain structure and function, it can also lead to oversimplifying the complex, polygenic nature of cognition and neurodegeneration. In addition, excluding individuals with cognitive difficulties may reduce variability and obscure the full spectrum of genotype effects, particularly their interactions with disease processes. However, we note that APOE is the largest genetic risk factor and the use of polygenic risk scores comes with its own pitfalls (Escott-Price and Schmidt, 2023; Leonenko et al., 2021; Stocker et al., 2023). Having only cross-sectional data barred us from testing whether longitudinal changes in brain activity in the same individuals can be related to APOE status, meaning our results may be biased by cohort effects (Raz and Lindenberger, 2011; Walhovd et al., 2023). We did not have direct data on neuropathology and or medicine intake. Finally, our sample included only White Europeans, whereas APOE might conceivably have differential effects in cohorts with other ancestral backgrounds.

In conclusion, we found strong evidence against APOE genotype-associated differences in processing naturalistic stimuli. We did not observe effects of APOE allelic status in pre-registered analyses of event boundary–related changes in univariate activity or connectivity. Moreover, exploratory analyses of average functional connectivity among DMN regions and whole-brain system segregation failed to detect differences between ε4 carriers and ε3/ε3 homozygotes. We speculate that APOE allelic status does not affect everyday cognition and functional brain connectivity in the absence of neuropathology relating to AD or other disease processes. Future work is needed to better understand the brain mechanisms through which particular variants of APOE increase the risk of developing AD.

## Supplemental Material

sj-docx-1-bna-10.1177_23982128251314577 – Supplemental material for No effect of apolipoprotein E polymorphism on MRI brain activity during movie watching Supplemental material, sj-docx-1-bna-10.1177_23982128251314577 for No effect of apolipoprotein E polymorphism on MRI brain activity during movie watching by Petar P. Raykov, Jessica Daly, Simon E. Fisher, Else Eising, Linda Geerligs and Chris M. Bird in Brain and Neuroscience Advances
