# Supplementary material for: No effect of apolipoprotein E polymorphism on MRI brain activity during movie watching
Source: Brain Neurosci Adv. 2025 Jan 31;9:23982128251314577. doi: 10.1177/23982128251314577 (PMC11783505; doi:10.1177/23982128251314577)
Supplement: sj-docx-1-bna-10.1177_23982128251314577 – Supplemental material for No effect of apolipoprotein E polymorphism on MRI brain activity during movie watching [file sj-docx-1-bna-10.1177_23982128251314577.docx]

# Supplementary Materials

## Classification based on connectivity matrices

Here we ran exploratory post-hoc analyses on the full coactivation matrices across all DMN sub-regions. Full matrices are shown in Figure 4 in the main manuscript. For each subject we extracted the low off-diagonal value of the full coactivation matrix (34x34), resulting in a 561 element feature vector. The pattern of coactivation was used as an input to a support vector machine classifier (Cortes & Vapnik, 1995) where we tried to classify participant’s genotype, whilst ignoring age by genotype interactions. The analyses utilised multivariate information that may be present in the coactivation pattern but might have been missed by focusing on a smaller number of posterior DMN regions. Given we did not have an equal number of ε3 and ε4 carriers we used balanced accuracy metric (e.g., see Thölke et al., 2023). We used a linear kernel SVM with 10 splits of stratified K-fold cross-validation. To test the significance of the classifier we permuted the genotype labels and ran the classification 5000 times. Our classification analyses could not robustly classify a participant’s genotype based the coactivation patterns.


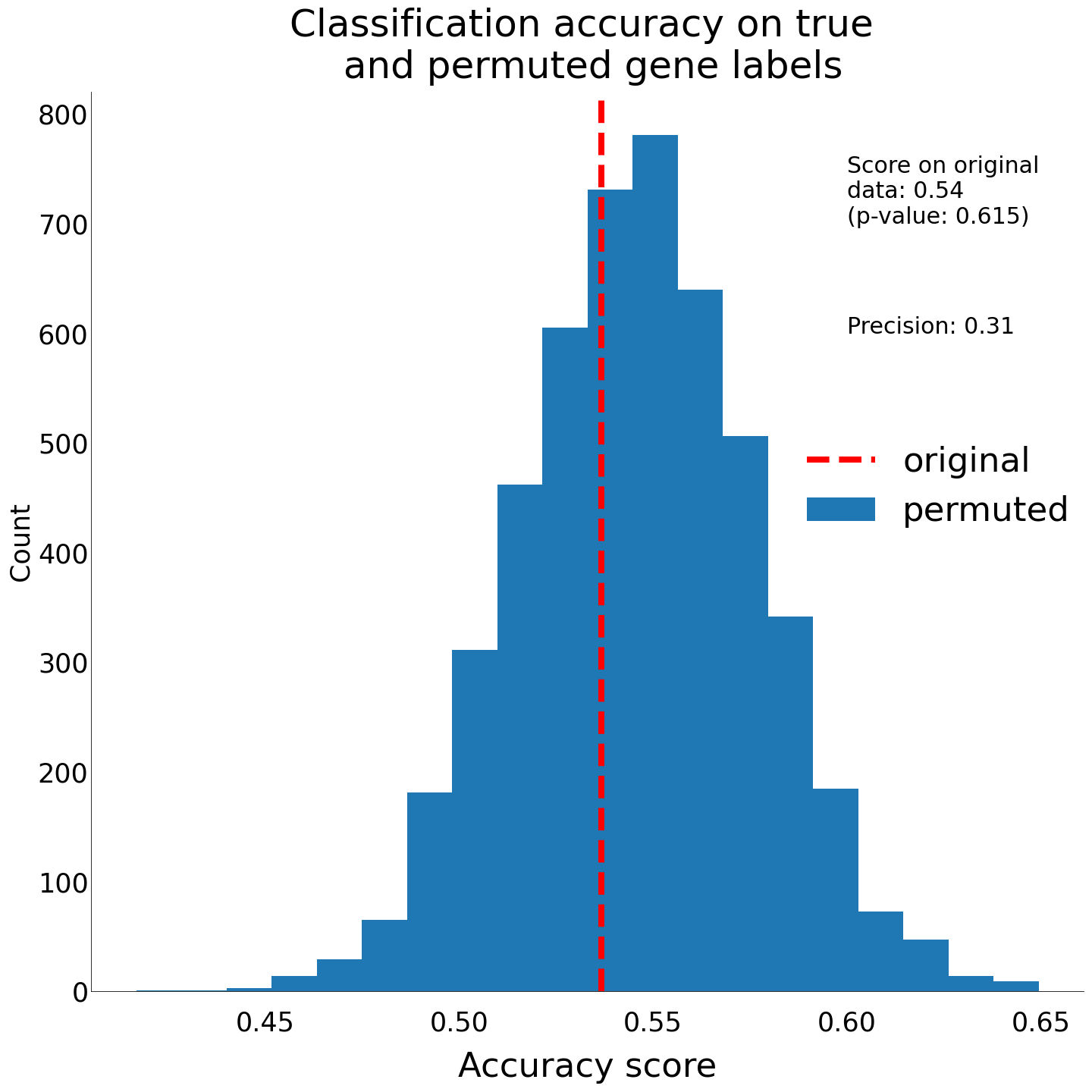


**Supplementary Figure 1.** Classification accuracy. We could not classify participant’s genotype based on their DMN connectivity pattern during event boundaries. The figure shows the histogram of cross-validated accuracies on permuted labels and the original accuracy shown in a dashed line.

## Functional Connectivity in DMN.

Previous reports have often examined average functional connectivity across DMN regions in resting state. Given that our main analyses focused on functional coactivation during event boundaries, we wanted to also examine genotype effects of functional connectivity using the whole duration of the movie. We computed functional connectivity across all pairs of 748 regions as defined by Geerligs et al., (2015). We then performed mean regression (MR) of the average connectivity across all nodes to account for vascular effects of aging (Geerligs et al., 2017; Yan et al., 2013). We then computed average functional connectivity among all pairs of the 87 DMN regions (see Supplementary Fig. 3), resulting a single value per participant. We fitted the same linear regression models we computed in the main report.

We did not find any genotype differences in average DMN connectivity measured during the movie task (see Supp. Fig. 2). Main effect of APOE (ß = 0.001 ± 0.002; t = 0.31, p = 0.75, BF_01_ = 22.95), Linear age by APOE interaction (ß = 0.004 ± 0.002; t = 1.96, p = 0.05, BF_01_ = 3.31), Quadratic age by APOE interaction (ß = 0.0003 ± 0.002; t = 0.15, p = 0.88, BF_01_ = 22.17).


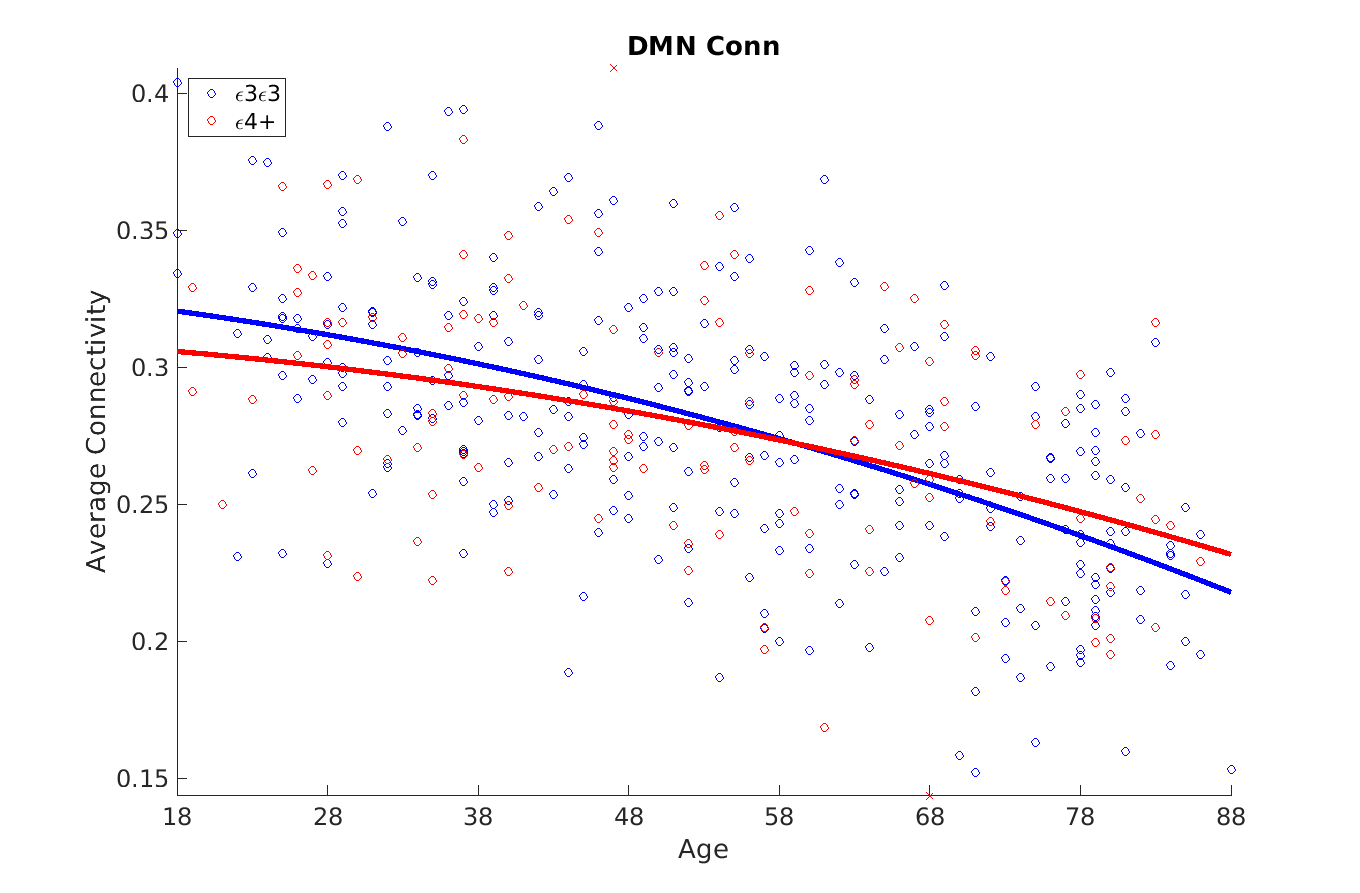


**Supplementary Figure 2** Average Functional connectivity across APOE carriers. We found no genotype difference in DMN functional connectivity measures computed across the whole duration of the movie.

## Functional System Segregation

We compared whether global measure of functional connectivity may show differences across genotypes. We computed functional system segregation as part of a previous report (see for details, Raykov et al., 2023). Briefly, this was a measure of the average within-network connectivity minus between-network connectivity, as a proportion of the within-network connectivity. This measure was computed during the whole duration of the movie across the whole brain using the Craddock atlas (2012) and age representative networks (see Supp. Fig. 2) defined based on a consensus partitioning algorithm applied to the Cam-CAN dataset (Geerligs et al., 2015; Lancichinetti & Fortunato, 2012). We focused on segregation in ‘associative’ brain networks thought to be associated with high-level cognition. Pre-computed SyS measures are available on osf (<https://osf.io/bq3a7/>). Note as previous reports we computed SyS measure after regressing out the global signal to account for vascular effects with age.

We did not find any genotype differences in SyS measured during the movie task (see Supp. Fig. 3). Main effect of APOE (ß = 0.003 ± 0.002; t = 1.03, p = 0.30, BF_01_ = 17.54), Linear age by APOE interaction (ß = 0.004 ± 0.003; t = 1.63, p = 0.10, BF_01_ = 7.54), Quadratic age by APOE interaction (ß = 0.004 ± 0.002; t = 1.37, p = 0.17, BF_01_ = 11.02).


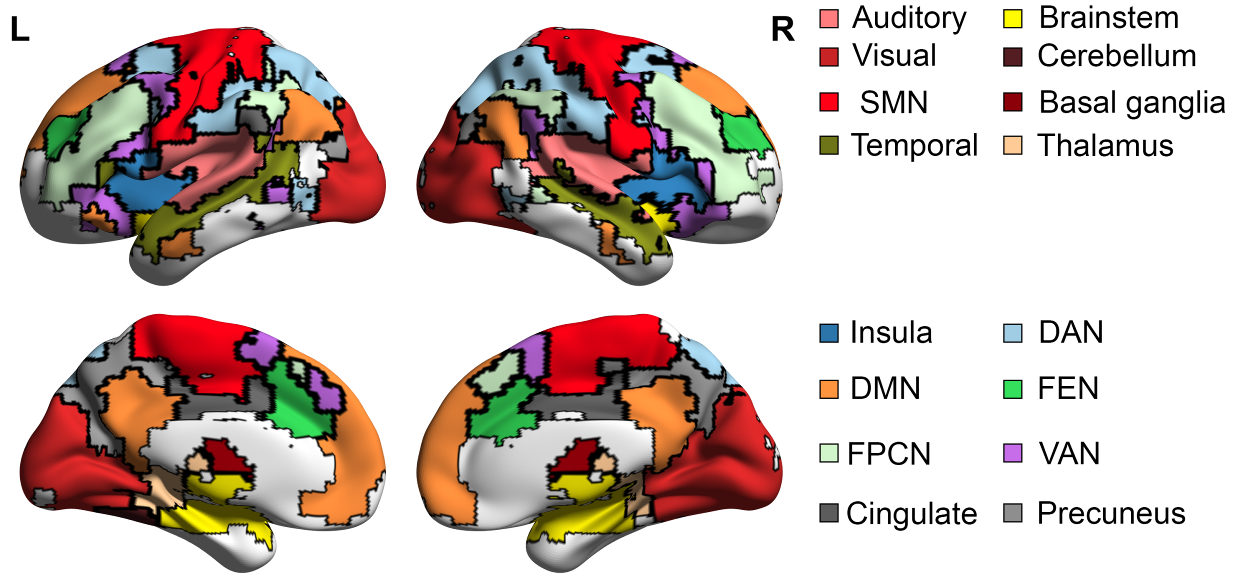


**Supplementary Figure 3** Craddock Networks. The plot shows the age-representative networks created in Geerligs et al., (2015) from the same data, starting with the Craddock brain parcellation. Following Chan et al. (2014), SyS measures excluded the non-associative networks (shown at top of legend). We focused our analyses on associative networks: Insula, DAN, DMN, FEN, FPCN, VAN, Cingulate and Precuneus (shown at bottom of legend).


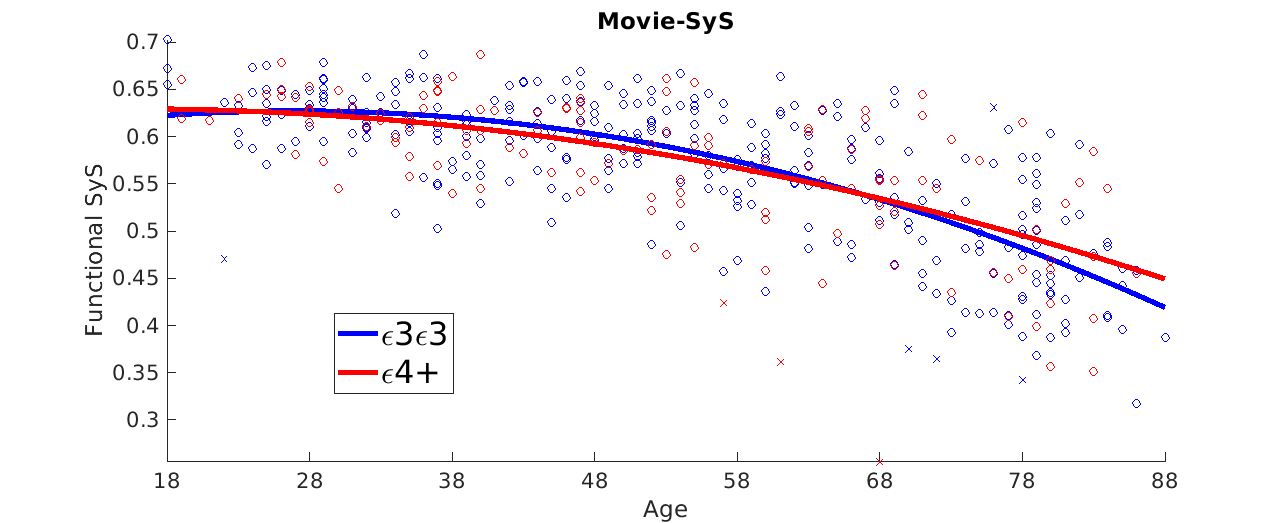


**Supplementary Figure 4.** Functional system segregation across APOE carriers. We found no genotype difference in movie SyS measures.
